# Urine and occlusion in the pathogenesis of lichen sclerosus: insights from a case of post-urethrectomy remission

**DOI:** 10.1093/skinhd/vzaf028

**Published:** 2025-04-16

**Authors:** Sachini Mendis, Georgios Kravvas, Richard Watchorn, Christopher B Bunker

**Affiliations:** Department of Dermatology, University College London Hospitals NHS Foundation Trust, London, UK; Department of Dermatology, University College London Hospitals NHS Foundation Trust, London, UK; Department of Dermatology, University College London Hospitals NHS Foundation Trust, London, UK; Department of Dermatology, University College London Hospitals NHS Foundation Trust, London, UK

## Abstract

We report the case of a 58-year-old uncircumcised man with male genital lichen sclerosus (MGLSc) achieving complete remission following urethrectomy and ileal conduit formation for urothelial carcinoma. The patient presented with typical clinical features of MGLSc. Initial treatment with ultrapotent topical corticosteroids provided only moderate improvement. Circumcision was planned but delayed due to the diagnosis of bladder and ureteral carcinomas. Postsurgery, urine was diverted through an ileal stoma, and the urethral meatus was sealed. Remarkably, despite cessation of topical treatments, the patient’s penile MGLSc showed complete resolution, with no residual active disease over a 4-year follow-up period. However, peristomal lichen sclerosus developed, emphasizing the role of occlusive urinary exposure in disease pathogenesis. This case supports the hypothesis that occlusive exposure to urine is a critical driver of MGLSc. The resolution of penile MGLSc following urethrectomy and the emergence of peristomal lichen sclerosus underscore the pathogenic importance of chronic urine contact. Key evidence includes the anatomical distribution of lesions, high prevalence of postmicturition dribbling, and resolution of MGLSc after circumcision or urethral isolation. This case highlights the interplay between urinary exposure, occlusion and epithelial susceptibility in MGLSc, and reinforces the necessity of addressing these factors in management. Further research into preventive strategies and targeted treatments for MGLSc and related conditions is warranted.

## Teaser

We present a case of male genital lichen sclerosus (MGLSc) achieving complete remission following urethrectomy and ileal conduit formation for urothelial carcinoma, highlighting the critical role of urinary occlusion in disease pathogenesis. The subsequent development of peristomal lichen sclerosus reinforces the link between chronic urine exposure and inflammation. This case provides unique insights into MGLSc management and supports strategies aimed at reducing urinary contact.

**What is already known about this topic?**

- Male genital lichen sclerosus (MGLSc) is strongly associated with occlusive exposure to urine, particularly in uncircumcised men.
- Resolution of MGLSc is commonly observed after circumcision or other interventions reducing urine contact.
- Peristomal lichen sclerosus is a recognized entity caused by chronic urine exposure at stoma sites.

**What does this study add?**

- This study highlights complete remission of MGLSc after urethrectomy and ileal conduit formation, demonstrating the role of ­eliminating urine exposure.
- The development of peristomal lichen sclerosus highlights urinary occlusion as a critical factor in the pathogenesis of lichen ­sclerosus.

## Case presentation

We report a case of male genital lichen sclerosus (MGLSc) where clinical remission was achieved after complete urethrectomy and ileal conduit formation.

**Figure 1:**
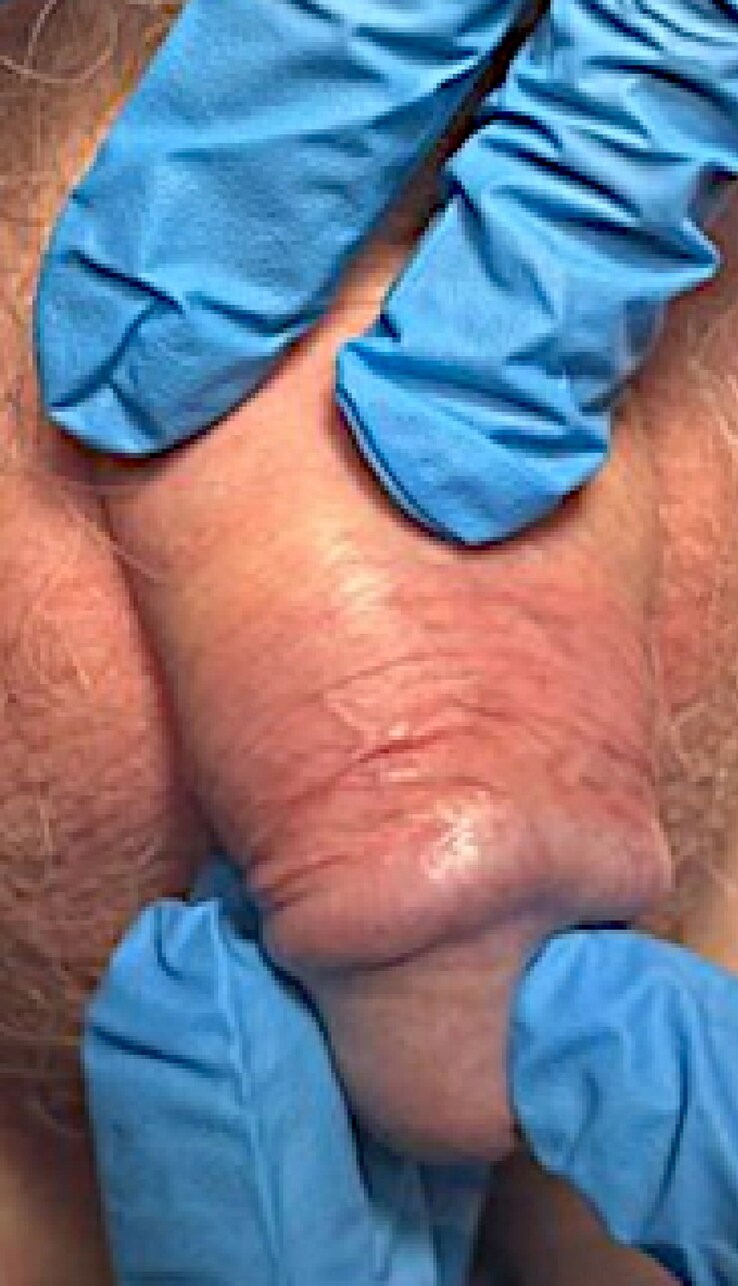
Active male genital lichen sclerosus prior to urethrectomy. Clinical photograph illustrating male genital lichen sclerosus (MGLSc) with active inflammation and chronic architectural changes prior to the urethrectomy. The image demonstrates areas of lichenoid inflammation, waxy pallor (sclerosis), loss of the coronal sulcus and constrictive posthitis with a prominent sclerotic band.

A 58-year-old uncircumcised male patient who formerly smoked presented to our tertiary male genital dermatology clinic in 2018 with penile erythema, itching and foreskin tightness. On examination, findings included an etiolated glans, trans-­coronal adhesions, and subtle erythematous-to-violaceous macular inflammation of the ventral frenulum and coronal sulcus (Figure 1). A firm clinical diagnosis of MGLSc was established. Initial management consisted of soap avoidance, skin barrier preparations and a 4-week course of clobetasol propionate ointment. Despite consistent adherence, the treatment achieved only moderate improvement and failed to provide a cure, leading to the recommendation of circumcision.

However, before circumcision could be performed, the patient was diagnosed with carcinoma of the bladder and right ureter (G3 pT1). He underwent robotic radical cystoprostatectomy, right nephroureterectomy, pelvic lymph node dissection and ileal conduit formation. The urine outflow was diverted to the ileal stoma, and the urethral meatus was sealed. Postsurgery, the patient discontinued the use of topical corticosteroids and barrier preparations for the glans and foreskin.

**Figure 2:**
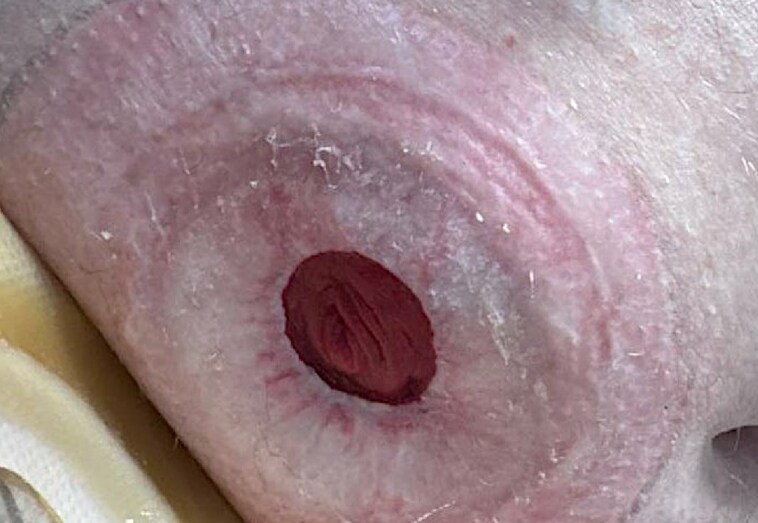
Development of lichen sclerosus (LSc) around the stoma site following urethrectomy. Clinical photograph demonstrating erythematous and scarring changes characteristic of LSc surrounding the stoma site. These features developed following urethrectomy and underscore the irritant effects of urine, a key factor in the pathophysiology of LSc.

**Figure 3:**
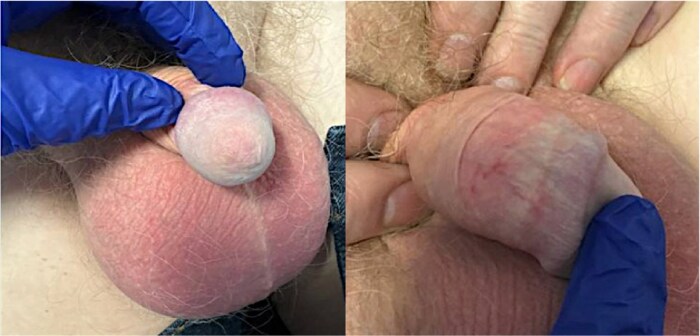
Remission of active male genital lichen sclerosus (MGLSc) following urethrectomy. Combined clinical photographs illustrating the remission of active lichen sclerosus (LSc) from the penis following urethrectomy. The images demonstrate features of burned-out LSc, including etiolation of the glans and residual scarring. While all signs of active inflammation have resolved, erythematous changes persist due to chronic LSc-driven telangiectasia. The cessation of inflammation highlights the removal of urine, a key factor in the pathogenesis of LSc, achieved through urethrectomy. Note the obliteration of the urethral meatus secondary to the removal of the urethra.

Two months after surgery, erythematous and scarring changes were noted around the stoma site (Figure 2). A clinical diagnosis of peristomal lichen sclerosus (LSc) was made, and treatment with barrier preparations and topical corticosteroids was initiated with good results. Since then, no residual signs of active MGLSc on the penis could be elicited, despite the patient remaining uncircumcised and having ceased topical treatments (Figure 3).

The patient has been under annual surveillance since, and the penile LSc remains quiescent.

## Discussion

LSc is an acquired, chronic, inflammatory skin disorder that predominantly affects the anogenital region. In male patients, it typically presents as waxy white, often atrophic patches and plaques. Purpura, telangiectasias or ecchymotic changes may also occur in the affected areas. Other common features include constrictive posthitis, lichenoid or zoonoid inflammation, thinning, and sclerosis of the skin, which can result in scarring, loss of elasticity, adhesions and architectural changes, such as the obliteration of the coronal sulcus. The frenulum is frequently affected, becoming fibrotic or, in some cases, resorbed.^1–3^

If left untreated or inadequately managed, MGLSc can lead to significant complications, including phimosis; it may involve the urethra causing stenosis; it is associated with differentiated penile intraepithelial neoplasia (dPeIN) and frank squamous carcinoma of the penis (PeSCC).^2,4,5^ More recently, emerging evidence has also suggested a potential link between MGLSc and the development of genital melanoma.^6–8^

MGLSc predominantly affects uncircumcised men and is strongly associated with postvoiding micro incontinence.^9^ It is proposed that in these individuals, after the foreskin is repositioned following urination, small droplets of residual urine leak from the urethral meatus and accumulate between the apposed mucosal surfaces of the glans and prepuce. This occlusive environment, combined with as-yet-undefined epithelial susceptibility factors, contributes to chronic inflammation and subsequent sclerosis.^10–12^

The presence of hypospadias, or interventions that can lead to postvoiding incompetence of the naviculomeatal valve, such as urological procedures and genital piercing, pose a recognized risk factor. Obesity is another important risk factor, not only as a systemic driver but also as a local mechanical contributor to the pathogenesis of MGLSc. Importantly, in circumcised men, obesity can lead to a ‘buried penis’ and ‘pseudoforeskin’ formation, where excess suprapubic fat and redundant penile skin form folds that recreate the occlusive environment seen in uncircumcised men. These represent the necessary conditions for urine trapping and prolonged tissue exposure, facilitating chronic irritation and inflammation.^6,8^

A growing body of evidence strongly supports the ­hypo­thesis that MGLSc is primarily driven by occlusive exposure to urine, and this case further strengthens that argument. Key evidence underpinning this hypothesis includes

(i) the anatomical distribution of LSc lesions, which aligns with areas of occlusive contact with urine;
(ii) the high prevalence of post-micturition dribbling in men with MGLSc;
(iii) the characteristic resolution of MGLSc following circumcision;
(iv) the anatomical abnormalities of the naviculomeatal fossa that are frequently seen in men with MGLSc; these impair its function as a low-pressure valve, thereby allowing urine leakage into the potential balanopreputial space;
(v) the increased risk of LSc following urological instrumentation or genital piercing; as these procedures may similarly impair the function of the naviculomeatal fossa as a low-pressure valve, promoting urine leakage into the balanopreputial space;
(vi) the sparing of the perianal region in MGLSc, unlike female genital LSc, due to the protective shielding provided by the foreskin and scrotum against urinary exposure; and
(vii) the recurrence of MGLSc in circumcised men when a pseudo- or neo-foreskin forms post-circumcision, associated with obesity; and
(viii) molecular evidence pointing to epithelial stress as the initiating event.^2,10,11,13,14^

Peristomal LSc has been increasingly recognized as a distinct manifestation of LSc, likely driven by chronic ­occlusion and exposure to irritants around the stoma site. Shim *et al*. reported cases of LSc developing in association with perineal urethrostomy, attributing its pathogenesis to the persistent contact of urine with peristomal skin, exacerbated by occlusion from stoma appliances.^15^ Similarly, Weng and Charles-Holmes described cases of LSc affecting colostomy sites, suggesting that the combination of mechanical irritation, occlusion and exposure to faecal material could act as triggers for disease development.^16^ Finally, Al-Niaimi and Lyon further highlighted the pivotal role of occlusion and urinary exposure in peristomal LSc, documenting cases where stoma appliances and chronic urinary contact created a localized environment conducive to inflammation and ­sclerosis.^17^

This case highlights the role of urinary occlusion in the pathogenesis of MGLSc and demonstrates a unique instance of remission following urethrectomy and ileal conduit formation.

## Data Availability

No new data were generated or analysed in support of this research.
